# Alterations of brain gray matter volume in children with obstructive sleep apnea

**DOI:** 10.3389/fneur.2023.1107086

**Published:** 2023-05-16

**Authors:** Chenyi Yu, Yuchuan Fu, Yi Lu, Yinyin Huang, Fangfang Chen, Jiayun Wei, Lingling Li, Janet Akoto Ampadu, Yu Wang, Weikun Zheng, Changcan Jiang, Weiyuan Li, Su Lui, Xiaohong Cai

**Affiliations:** ^1^Department of Pediatrics, The Second Affiliated Hospital and Yuying Children's Hospital of Wenzhou Medical University, Wenzhou, China; ^2^Department of Radiology, The Second Affiliated Hospital and Yuying Children's Hospital of Wenzhou Medical University, Wenzhou, China; ^3^Department of Otorhinolaryngology, The Second Affiliated Hospital and Yuying Children's Hospital of Wenzhou Medical University, Wenzhou, China; ^4^Department of Pneumology, Yuxi Children's Hospital, Yuxi, China; ^5^Department of Radiology, West China Hospital of Sichuan University, Chengdu, China

**Keywords:** obstructive sleep apnea, children, gray matter volume, MRI, Das-Naglieri cognitive assessment system

## Abstract

**Objective:**

Obstructive sleep apnea (OSA) seriously affects the children's cognitive functions, but the neuroimaging mechanism of cognitive impairment is still unclear. The purpose of our study was to explore the difference in brain local gray matter volume (GMV) between children with OSA and non-OSA, and the correlation between the difference regions of brain gray matter volume and cognitive, the severity of OSA.

**Method:**

Eighty-three children aged 8–13 years were recruited in our study, 52 children were diagnosed as OSA by polysomnography, and 31 as the non-OSA. All the subjects were underwent high-resolution 3-dimensional T1-weighted magnetic resonance images. The voxel-based morphometry (VBM) was be used to analyse the local GMV. The Das-Naglieri cognitive assessment system (DN: CAS) was used to assess the subjects' cognitive. The difference of local GMV between the two groups was analyzed by two-sample *T*-test. The PSG variables and the scores of DN: CAS between the OSA group and non-OSA group were compared by independent samples *t*-tests. Pearson correlation was used to calculate the association between the difference areas of gray matter volumes in brain and DN: CAS scores, obstructive apnea/hypopnea index (OAHI, an index of the severity of OSA).

**Results:**

The gray matter volume of the right Middle Frontal Gyrus (MFG_R) in OSA children were larger than the non-OSA children, and the OSA children had lower scores of the Word Series in DN: CAS. There was negative correlation between the scores of Expressive Attention in DN: CAS and the gray matter volume of the right middle frontal gyrus, and it was no significantly correlation between OAHI and the gray matter volume of the right middle frontal gyrus.

**Conclusion:**

Our results suggest that the development of gray matter volume in frontal cortex, which associated with attention, were sensitive to the effects of OSA, provides neuroimaging evidence for cognitive impairment in children with OSA.

## Introduction

Obstructive sleep apnea (OSA) is a common disorder among children, with prevalence ranging from 1% to 5% (1, 2). It is characterized by partial or complete obstruction of the upper airway during sleep, leading to nocturnal hypoxemia, and sleep fragmentation (3, 4). Previous studies have revealed that OSA can lead to cognitive impairment in children, including learning disabilities and inattention (5–7).

Intermittent hypoxia was the primary characteristic of OSA, previous animal studies showed that it caused the changes of brain metabolic and structural. Li et.al found that Intermittent hypoxia could lead to neuronal apoptosis in frontal cortex neurons of rat by increased the expression of cysteinyl aspartate specific protease (caspase-8) (8). Zeng et al. (9) reported that intermittent hypoxia could change the neuron ultrastructural in the frontal lobe cortex of mice, such as swelling of chondriosomes, degeneration of neural sheath and karyopyknosis of nucleus (9). Kheirandish et al. (10) aslo found that the dendritic branches and the number of dendritic spines in frontal cortex was decreased in developing rats exposed to intermittent hypoxia for 14 days starting at postnatal 10 days (10).

Multiple meta-analyses have investigated the effects of obstructive sleep apnea (OSA) on brain gray matter in adults using Voxel-based Brain Morphometry Studies. Huang et al. reported a decrease in gray matter volume in the bilateral anterior cingulate/paracingulate gyri, hippocampus/parahippocampal gyrus, the orbital frontal cortex, and left cerebellum VI in adults with OSA (11). Shi et al. (12) found reductions in gray matter in the bilateral anterior cingulate/paracingulate gyri, left cerebellum (lobules IV/V and VIII), bilateral superior frontal gyrus (medial rostral part), right middle temporal gyrus, and right premotor cortex in adults with OSA (12). Weng et al. (13) demonstrated local gray matter reduction in the bilateral parahippocampus, the right superior frontal, and left middle temporal gyri in adults with OSA (13).

These meta-analyses suggest that OSA-related damage to brain gray matter in adults occurs primarily in the frontal and limbic regions, which are associated with cognitive functions. However, it remains unclear whether the patterns of brain gray matter injury in children with OSA are consistent with those observed in adults, and the number of studies investigating the effect of OSA on gray matter structure in children is relatively low.

Macey's team found that the GMV of bilateral prefrontal cortices was reduced in OSA children and the cortical thickness of right medial prefrontal areas was increased, meanwhile there was no significant association between the changes of the gray matter structure and the cognitive test scores (14, 15). However, the great majority of control samples in these studies from the preexisting data, it might be contained OSA patients because these children were not assessed by PSG. Musso et al. (16) found the GMV increased in right frontal lobe in children with OSA (16), but cognitive function of these subjects were not been evaluated.

Das-Naglieri cognitive assessment system (DN: CAS) was explored by JP. Das based on Planning-Attention-Simultaneous-Successive (PASS) theory. PASS theory reconceives intelligence as a process-driven understanding of cognitive abilities. It is based on four cognitive processes: planning, attention, simultaneous processing, and successive processing. The PASS model was based on Luria's model of the higher cortical functions in human (17). PASS theory links the four cognitive processes with the particular regions of the brain, such as the units of simultaneous and successive have been link to occipito-temporoparietal junction and frontotemporal and perisylvian opercular regions (17). At present, studies in children with OSA mostly used the Wechsler Intelligence Scale for Children (WISC) to assess the subjects' cognitive function. Compared to WISC, the score of DN: CAS was measured not only by assessing the accuracy of completing a task, but also by evaluating the time they take to complete the task. Rostami et al. reported that DN: CAS scores seemed more helpful in differentiating the children with attention deficit hyperactivity disorder (ADHD) and specific learning disabilities when compared with WISC (18). Another study showed that DN: CAS was more sensitive than the WISC for detecting the selective neurocognitive weaknesses in children with moyamoya disease (19). Given that previous researches, our study used DN: CAS to assess cognitive function of the subjects.

The childhood is the peak period of brain gray matter development (20, 21), how the impairment pattern of the brain gray matter development affected by OSA remains unclear. It is still to be explored whether there was correlation between the difference areas of the brain gray matter volume and cognitive, the severity of OSA. For these purpose, firstly we compared the difference areas of the brain gray matter volume between the OSA children and non-OSA children. Then, we used the obstructive apnea-hypopnea index (OAHI) of PSG recordings to represent OSA severity, DN: CAS was used to assess the cognition in children, we analyzed the correlation between the difference areas of brain gray matter volume and cognitive function, OAHI.

## Materials and methods

### Subjects

This study carried out with the permission of the hospital ethics committee of the second affiliated hospital Wenzhou medical university. All the subjects' guardians wrote informed consent before study participation. The subjects were recruited in our Pediatric Sleep Medicine Department from March 2018 to October 2020. The exclusion criteria were as follows: (1) psychiatric or neurologic illness; (2) history of brain tumors, trauma, and infections; (3) suffering from cardiovascular diseases, neuromuscular diseases, or defined genetic syndromes; (4) abnormal blood pressure, blood fat, and glucose; (5) being with any known acute or chronic illness; and (6) undergone treatment with drugs and surgery; (7). The Full-Scale IQ < 70 assessed by Wechsler Intelligence Scale for Children- IV; (8) intolerance to MRI examination.

### Polysomnography

Standard overnight Polysomnography (PSG) was performed using a computerized PSG system (Alice5 Koninklijke Philips N.V. Philips Respironics, Murrysville, PA, United States), which recorded polysomnograms lasting more than seven hours.

All sleep data were calculated and evaluated by a trained PSG technician. Sleep was divided into wakefulness and sleep stages based on multiple channels of electroencephalogram signals (C3/A2, C4/A1, O1/A2, O2/A1), eye movement, submental electromyogram, electrocardiogram, pulse oximetry, thoracic and abdominal respiratory movements, and body position. Respiratory events were defined as episodes of apnea or hypopnea during sleep, which could be further classified as obstructive apnea, central apnea, mixed apnea, or obstructive hypopneas based on changes in thoracic and abdominal respiratory movements, respiratory rate, and amplitude. Obstructive apnea-hypopnea index (OAHI) was the total number of obstructive apneas, obstructive hypopneas, and mixed apneas per hour of total sleep time. In the current study, obstructive apnea/hypopnea index (OAHI) >1 events/h was the criterion for diagnosis of OSA. Children with an OAHI of ≤ 1 event/h were diagnosed as non-OSA, while those with an OAHI of >1 event/h were diagnosed with OSA. Among the non-OSA children, those with a clinical history of snoring were diagnosed with primary snoring, while those without a snoring history and no snoring observed during PSG were considered healthy children. In the current study, the severity of OSA was classified according to OAHI, as mild OSA was defined as an OAHI of 1 to 5 events/h, moderate OSA was defined as an OAHI>5 to ≤10 events/h, and severe OSA was defined as an OAHI>10 events/h (22).

### Neurocognitive assessments

Cognitive function was assessed using the Das-Naglieri cognitive assessment system (DN: CAS) in the next morning, which had been administered by an experienced psychotherapist. The DN: CAS (23) includes four sub-scales: Planning, Attention, Simultaneous Processing and Successive Processing, each scale was consisted of three sub-projects. Planning assessment was used to evaluate the efficiency in task solving, which includes three subsets: Matching Numbers, Planned Codes and Planned Connections. Attention mainly assesses the ability to selectively attends to a particular stimulus, which includes three subsets: Expressive Attention, Number Detection and Receptive Attention. Simultaneous processing mainly assesses the ability to integrate the segments in the item by abstract thinking and logical perception, including Non-verbal Matrices, Verbal-Spatial Relations and Figure Memory. Successive Processing mainly assesses the ability to understand or repeat auditory information that is presented in specific order, it contains three subsets: Word Series, Sentence Repetition and Sentence Questions.

### Magnetic resonance imaging acquisition

MRI data were collected from all children within 2 days of the sleep study using a 3.0 Tesla scanner (Discovery 750, GE Healthcare, USA) with a 32-channel phased-array head coil. Participants were required to keep lying still and close their eyes, a noise reduction earplug was used to protect the auditory system and a sponge pad to reduce head movement. All the subjects were underwent high-resolution 3-dimensional T1-weighted magnetic resonance images. The acquisition parameters were as follows: slices = 188, field of view (FOV) = 256 mm × 256 mm, echo time (TE) = 3.4 ms, repetition time (TR) = 7.7 ms, flip angle (FA) = 9°, matrix size = 256 × 256, slice thickness = 1 mm, no gap.

### Magnetic resonance imaging data analysis

For brain image assessments, we used the Computational Anatomy Toolbox (CAT12) (http://dbm.neuro.uni-jena.de/vbm) as implemented in the SPM12 software (http://www.fil.ion.ucl.ac.uk/spm/) to perform voxel-based morphometry (VBM). Data were processed using the following steps: (1) by linearly registering the images to the International Consortium for Brain Mapping (ICBM152) template, the images were normalized to standard space; (2). A study-specific template for children was created using DARTEL; (24). (3) The study data were then normalized to this custom anatomical template using segmentation of gray matter, white matter, and cerebrospinal fluid. (4) An 8 × 8 × 8 mm (half-height full width) Gaussian smoothing kernel was used to improve the signal-to-noise ratio of the modulated normalized gray matter graph (25).

### Statistical analyses

The continuous variables were presented as the mean ± standard deviation. The Kolmogorov-Smirnov normality test was used to examine the distribution of data. Chi-square tests were performed for gender. Independent samples *t*-tests were applied to examine differences in age, Body mass index (BMI), PSG results and Each DN: CAS subset between two groups. Statistical analyses were conducted using the statistical analysis software package SPSS (Version 26.0, IBM, Inc., USA) with a significance level of *P* < 0.05.

Two-sample *t*-test performed to identify differences in whole brain gray matter volume, controlling for age, sex, and BMI as covariates. Whole-brain multiple comparisons were corrected using a Gaussian Random Field (GRF) correction with a cluster-defining threshold of *P* < 0.001. Pearson correlations were performed between the difference area of brain gray matter volumes and the scores of DN: CAS, OAHI.

## Results

### Demographic and clinical information

Eighty three children were enrolled in our study, fifty-two children were diagnosed with OSA, and thirty-one children for the non-OSA group, in which thirty-three children had mild OSA, fourteen had moderate OSA, five had severe OSA, ten children as primary snoring, and twenty-one as healthy children. There were no statistically difference in age and gender between the OSA group and non-OSA group. BMI (*t* = −2.534, *P* < 0.05) and OAHI (*t* = −4.550, *P* < 0.01) in OSA group had significantly higher than non-OSA group, the scores of Word Series subtest of DN: CAS had significantly lower in children with OSA compared to the non-OSA group (*t* = 2.882, *P* < 0.05). The demographic and clinical details for the subjects were presented in Table 1.

**Table 1:** Demographic and clinical characteristics for the subjects.

	**OSA group (*N =* 52)**	**Non-OSA group (*N =* 31)**	**χ^2^/t**	***P*** **-value**
Age (years)	8.77 ± 1.436	9.13 ± 1.176	1.178	0.242
**Gender**				
Male	27 (51.9%)	14 (45.2%)	0.355	0.551
Female	25 (48.1%)	17 (54.8%)		
BMI (kg/m^2^)	18.87 ± 4.05	16.75 ± 2.94	−2.534	0.013^*^
OAHI (events/h)	7.05 ± 10.34	0.52 ± 0.27	−4.550	0.000^*^
Planning	26.42 ± 5.66	27.58 ± 5.03	0.939	0.350
Matching numbers	10.31 ± 2.74	11.03 ± 2.55	1.195	0.235
Planned codes	7.60 ± 1.99	8.03 ± 1.80	1.026	0.308
Planned connections	8.52 ± 2.54	8.52 ± 2.29	−0.006	0.996
Simultaneous	38.96 ± 5.75	40.45 ± 5.59	1.154	0.252
Non-verbal Matrices	13.31 ± 2.48	13.42 ± 2.20	0.207	0.837
Verbal-spatial relations	11.90 ± 2.84	12.81 ± 2.97	1.378	0.172
Figure memory	13.81 ± 2.37	14.23 ± 2.75	0.732	0.466
Attention	29.62 ± 5.90	31.26 ± 5.11	1.289	0.201
Expressive attention	10.52 ± 2.71	11.19 ± 3.240	1.018	0.312
Number detection	9.44 ± 2.69	10.35 ± 1.76	1.866	0.066
Receptive attention	9.63 ± 2.48	9.71 ± 2.12	0.141	0.889
Successive	35.48 ± 6.52	38.03 ± 6.03	1.774	0.080
Word series	16.35 ± 2.61	17.81 ± 1.97	2.882	0.005^*^
Sentence repetition	8.19 ± 2.27	8.58 ± 2.31	0.750	0.455
Sentence questions	10.94 ± 3.11	11.65 ± 3.03	1.006	0.317
Full scale	130.48 ± 18.11	137.32 ± 16.70	1.713	0.091

* *P* < 0.05. BMI, Body mass index; OAHI, obstructive apnea/hypopnea index.

### Differences in gray matter volume between groups

**Figure 1:**
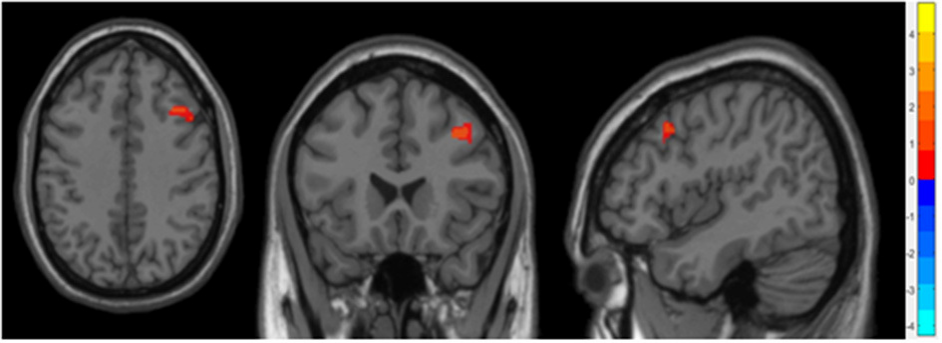
It showed the difference regions of GMV in slices with various directions between the OSA and non-OSA groups (*P* < 0.05, GRF corrected). The red clusters indicate a larger GMV for children with OSA than for controls.

Compared to the non-OSA group, the gray matter volume of the right Middle Frontal Gyrus (MFG_R) was increased in the children with OSA (*P* < 0.05, GRF correction; Table 2 and Figure 1).

**Table 2:** Brain regions showing significant differences in GMV.

**Conditions**	**Brain regions**	**Peak MNI**^**a**^ **coordinates**			**Cluster voxels**
		x	y	z	
PG > HG	the right Middle Frontal Gyrus	43.5	16.5	42	216

MNI^a^, Montreal Neurological Institute; GMV, gray matter volume; PG, patient group (OSA group); HG, healthy control group (non-OSA group).

### Correlation analysis

**Figure 2:**
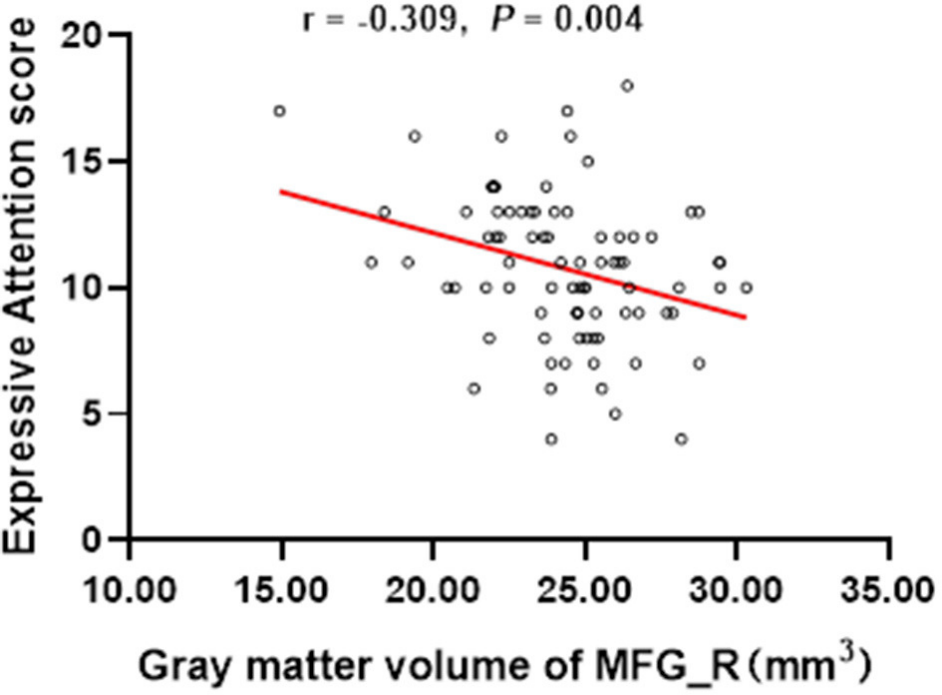
In the whole group, the expressive attention score was significantly negatively correlated with the gray matter volume of the right medial frontal gyrus (*r* = −0.309, *P* = 0.004). MFG_R, the right medial frontal gyrus.

The results showed that the gray matter volume of MFG_R was significantly negative association with the score of the Expressive Attention assessment in DN: CAS (*r* = −0.309, *P* = 0.004; Table 3 and Figure 2). There was no significant association between the gray matter volume of MFG_R and OAHI.

**Table 3:** Results of Pearson correlation analysis between the gray matter volume in MFG_R and the outcome of DN: CAS, OAHI in the whole group.

	**MFG_R**	
	***r***	***P*** **-value**
OAHI (events/h)	0.033	0.767
Planning	0.129	0.245
Matching numbers	0.133	0.230
Planned codes	0.050	0.656
Planned connections	0.102	0.360
Simultaneous	−0.090	0.420
Non-verbal Matrices	−0.092	0.406
Verbal-spatial relations	−0.070	0.527
Figure memory	−0.022	0.840
Attention	−0.112	0.312
Expressive attention	−0.309^**^	0.004
Number detection	0.179	0.106
Receptive attention	−0.074	0.507
Successive	−0.116	0.296
Word series	−0.059	0.595
Sentence repetition	−0.170	0.124
Sentence questions	−0.068	0.539
Full scale	−0.067	0.549

The symbol

** denotes *p* < 0.01.

MFG_R, the right medial frontal gyrus; OAHI, obstructive apnea/hypopnea index; DN: CAS, Das-Naglieri cognitive assessment system.

## Discussion

To our knowledge, this is the first study to evaluate the cognitive function in the OSA children using the DN: CAS and analyze the correlation between the difference areas of gray matter volume and the scores of the DN: CAS and OAHI. Our results showed a significant increase in gray matter volume in the right medial frontal gyrus (MFG_R) in the OSA group. Moreover, we observed a negative correlation between changes in gray matter volume in the right middle frontal gyrus (MFG_R) and Expressive Attention scores in DN: CAS. Accordingly, these findings might be provided neuroimaging evidence for cognitive impairment in childhood OSA.

In this study, the gray matter volume of the MFG_R was larger in children with OSA. Previous study about regional brain tissue integrity in OSA children assessed by entropy tissue texture reported that the entropy in the pre-frontal cortex was decrease in OSA children, and it indicated acute tissue injury in the prefrontal lobe (26). They speculated that it possibly reflecting the unique susceptibility of this brain area to the hypoxia-reoxygenation injury (26, 27). The primary pathophysiological characteristic of OSA were Intermittent hypoxia and reperfusion (28). Previous studies in rodent models found that intermittent hypoxia could cause the adaptability and responsiveness changes in brain such as altered the expression of brain aquaporin and increased brain water content, increased the neuronal branching of surviving neurons and the number of progenitors and newborn neurons, and astroglial hypertrophy and/or hyperplasia (29–32). We guessed those might be the reason for the changes of the gray matter volume. The result in our study about increased GMV in right frontal lobe was in agreement with the found by Musso et al. (16), but opposite with Philby et al. (14). Philby et al. (14) found that the GMV of the right prefrontal was reduced in OSA children (14), we guessed the possible reason was that the great majority of control samples in Philby's research were from the NIH-Pediatric MRI database, these samples were not assessed by PSG, it thus might contain OSA patient. Moreover, the findings from VBM studies may also be influenced by the selection of covariates and the relationships among covariates and outcome variables. Prior research has reported an association between changes in brain gray matter volume in adults with OSA and BMI (11, 33). In Philby's study, only age and gender were used as covariates when processing the data using VBM, whereas in our study, we additionally controlled for BMI as a covariate.

Correlation analysis indicated that the gray matter volume of the MFG_R was negative association with the expressive attention scores. Expressive attention is a subitem of attention assessment in DN: CAS, it mainly assessed the ability to inhibit interfering stimuli during the expression process (34). Previous research verified that the MFG_R played an important role in attention (35). Li et al. (36) found that the attention decline in adults with acute sleep deprivation were associated with the decreased functional connectivity between the MFG _ R and the precuneus in resting-state functional magnetic resonance imaging study (36). Sleep fragmentation caused by OSA could also lead to sleep loss. The change of the MFG_R might be provided neuroimaging evidence for attention decline in OSA children.

In our study, there was no correlation between the difference areas of brain gray matter volume and OAHI. Previous studies in childhood OSA have reported the similar results. Philby et al. (14) found no significant association between the change area of the brain gray matter volume and the Apnea Hypopnea Index (AHI, another index of the severity of OSA) (14). Musso et al. (16) found that there was no significantly difference in the GMV of frontal between the mild OSA group and the moderate/severe OSA group, they speculated the severity of OSA (measured by the AHI) wasn't correlate with the extent of gray matter volume alterations in frontal regions (16). However, Baril et al. (37) reported that the left thalamus gray matter volume was increased in adult with OSA, and it was positively correlated with the severity of OSA (AHI was a principal component) (37). The reason might be that in the populations of adults and children, the current clinical polysomnographic criteria for OSA diagnosis was significantly different. In pediatric cohort, moderate to severe OSA in children would be equivalent to mild or normal OSA in adults.

In terms of cognitive function, our study found that the OSA group had lower Word Series scores than the non-OSA (control) group. The Word Series task, a subtest of the successive processing, the administrator reads words at a speed of 1 s/word, and asks the subject to repeat them. Previous studies showed that the OSA was associated with impairment of procedural and verbal declarative memory (38, 39). Successive processing has been related to the cortical functions of the occipito-temporoparietal junction and frontotemporal and perisylvian opercular regions according to PASS theory (17). However, there was no difference in the above brain regions GMV between OSA and non-OSA groups in our study. Castronovo et al. observed that normal working-memory performance in OSA patients is associated with increased left fronto-lateral activity and reduced right fronto-lateral activity compared to controls (40). Based on this, we speculate that changes in cognitive function related to word series might be associated with alterations in functional brain networks that are linked to memory, rather than changes in local brain gray matter volume.

There are some limitations in our study. First of all, the small sample size was an issue that must be acknowledged. Secondly, a small percentage of the children in non-OSA group are the children with primary snoring and the limited number of severe OSA patients, which may lead to biased conclusions. Therefore, our experimental results need to be further confirmed by expanding the sample size and recruiting healthy children as the control group. Finally, our study was a cross-sectional study. How the brain gray matter development in OSA children evolves, and whether the damage of brain gray matter reversibility after treatment is unknown.

## Conclusion

The results of this study suggest that OSA affects the development of gray matter in children, which provides neuroimaging basis for the cognitive impairment of children with OSA. As a consequence, we advocate for the early detection of OSA and the timely intervention to minimize its effects on brain development in children, and to help optimize the repair and recovery of damaged brain tissue.

## Data availability statement

The raw data supporting the conclusions of this article will be made available by the authors, without undue reservation.

## Ethics statement

The studies involving human participants were reviewed and approved by the Hospital Ethics Committee of the Second Affiliated Hospital at Wenzhou Medical University. Written informed consent to participate in this study was provided by the participants' legal guardian/next of kin.

## Author contributions

XC and SL: experimental design. YF, YL, YH, and YW: MRI data collection. JW, LL, WL, and JA: sleep variables and clinical data collection. WZ and CJ: data curation. FC and CY: data analysis. CY and YF: manuscript preparation. All authors contributed to the article and approved the submitted version.
